# Emotional distress in the Chinese academic context: an integrative mixed-methods study of gratitude, savoring, and cognitive reappraisal

**DOI:** 10.3389/fpsyg.2026.1844831

**Published:** 2026-08-04

**Authors:** Xuemei Hou, Dunhui Zhan, Tan Lu, Heung Kou

**Affiliations:** Department of Education, Graduate School, Sehan University, Yeongam-gun, Republic of Korea

**Keywords:** emotional distress, gratitude, savoring beliefs, cognitive reappraisal, Chinese university students, mixed-methods

## Abstract

**Introduction:**

Chinese university students face escalating mental health challenges, yet the psychological mechanisms that protect against emotional distress remain inadequately understood. This study investigates whether cognitive reappraisal mediates the relationship between positive psychological resources (gratitude and savoring beliefs) and emotional distress in this population.

**Methods:**

A convergent parallel mixed-methods design was employed with 497 Chinese university students (49.9% female, mean age 20.3 years) recruited from three large public universities in Eastern China. Quantitative data were collected using the Gratitude Questionnaire-Six Item Form (GQ-6), Savoring Beliefs Inventory (SBI), Emotion Regulation Questionnaire (ERQ) reappraisal subscale, and Hopkins Symptoms Checklist-10 (HSCL-10). Structural equation modeling (SEM) with bootstrapping tested the mediation model. Semi-structured interviews (*n* = 20) were analyzed using thematic analysis to explore phenomenological experiences.

**Results:**

The SEM demonstrated robust fit (χ^2^/*df* = 1.80, *CFI* = 0.95, *RMSEA* = 0.041), revealing that gratitude and savoring significantly reduced emotional distress through partial mediation by cognitive reappraisal (indirect effects = −0.18 and −0.15, respectively), explaining 41% of the variance in emotional distress. Qualitative analysis identified three core themes: gratitude as a relational “anchor,” savoring as temporal “decentering,” and a self-reinforcing “benefit-finding loop.”

**Discussion:**

Findings support the broaden-and-build theory, demonstrating that positive resources facilitate adaptive emotion regulation by providing the cognitive flexibility necessary for effective reappraisal. The integration of quantitative and qualitative data reveals that gratitude and savoring do not merely coexist with stress but actively transform how students process academic adversity. These results underscore the importance of resource-primed regulation protocols in university mental health interventions.

## 1 Introduction

The mental health of university students has emerged as a global public health priority, with increasing rates of emotional distress characterized by symptoms of anxiety and depression (Jans-Beken et al., 2020; Wood et al., 2010). This issue is particularly acute within the higher education landscape of Mainland China, where students navigate an environment defined by intense academic competition, high parental expectations, and the persistent legacy of the “Gaokao” culture (Man and Jing, 2025; Zhang et al., 2022). Large-scale data from 106 Chinese universities (*N* = 41,620) show depression and anxiety detection rates of 9.8 and 15.5%, with 6.5% comorbidity (Han et al., 2025). Broader screening of 1,790 students finds higher rates: 38.7% depressive and 17.8% anxiety symptoms (Li et al., 2023). Across 15 universities, 24.54% of students have suboptimal mental health (Fan et al., 2024). Regarding resilience, post-pandemic data from 1,735 students indicate only moderate levels (*M* = 2.949/5; Sheng et al., 2025), and resilience is a strong negative predictor of distress (Fan et al., 2024). Chen et al. (2024) confirm that depression and anxiety are the primary mental health challenges facing college students. Despite clear need, help-seeking remains low, highlighting a critical gap in understanding what psychological resources allow some students to thrive under pressure while others struggle.

The urgency of this research lies in the escalating consequences of untreated emotional distress and low resilience among university students. Persisting problems lead to measurable academic, social, institutional, and economic costs. Individually, distressed students face higher risks of academic underachievement, course withdrawal, and dropout—depressive symptoms predict a 40–50% increase in dropout risk (Eisenberg et al., 2009). Socially, they withdraw from support networks, worsening isolation. Institutionally, universities face rising counseling demands, with some Chinese campuses reporting weeks-long wait times; delayed help can lead to maladaptive coping, including self-harm. Economically, premature departure or underperformance due to mental health struggles erodes human capital and workforce productivity. In the Chinese context, where families invest heavily in a single child, a student’s mental health crisis can destabilize the entire household. Thus, identifying protective mechanisms (gratitude, savoring, reappraisal) is a necessary step toward scalable, culturally sensitive interventions to prevent these cascading negative outcomes. Despite the prevalence of these stressors, many students exhibit remarkable resilience, suggesting that specific psychological resources may function as protective buffers against maladjustment. Among these resources, positive psychological constructs such as dispositional gratitude and savoring beliefs have gained significant attention for their role in promoting well-being (Bryant and Veroff, 2007; McCullough et al., 2002). However, while the inverse relationship between these positive resources and emotional distress is well-established, the underlying cognitive mechanisms through which they exert their protective influence remain insufficiently understood, particularly in non-Western academic settings.

Gratitude, defined as a generalized tendency to recognize and appreciate the benevolence of others and the positive aspects of one’s life, has been robustly linked to lower levels of depression and stress (McCullough et al., 2002; Wolfe, 2021). Similarly, savoring—the metacognitive capacity to attend to, appreciate, and enhance positive experiences—enables individuals to maintain emotional equilibrium by broadening their temporal perspective (Bryant, 2003; Gao et al., 2025). Theoretically, these traits do not merely coexist with negative affect; rather, they are thought to provide the “cognitive capital” necessary to transform how an individual processes adversity. According to the broaden-and-build theory, positive affective states expand an individual’s thought-action repertoire, thereby facilitating the development of durable personal resources such as adaptive emotion regulation (Fredrickson, 2001; Garland et al., 2010). Within this framework, cognitive reappraisal—an antecedent-focused strategy involving the reframing of a situation’s meaning to alter its emotional impact—stands out as a primary candidate for the mechanism linking positive traits to reduced distress (Gross and John, 2003).

Despite the conceptual alignment between positive resources and emotion regulation, several gaps persist in the literature. First, most studies have examined gratitude, savoring, and reappraisal in isolation or within purely quantitative frameworks that reduce complex psychological processes to abstract path coefficients (Hayes, 2018; Maxwell, 2012). Consequently, the lived experiences and phenomenological nuances of how students leverage a grateful or savoring mindset to reframe their stressors remain largely obscured. Second, while the protective roles of these constructs are documented in Western samples, the Chinese cultural context—which emphasizes relational harmony, filial piety, and “face”—presents a unique psychological landscape that may shape the enactment of these regulatory strategies in culturally specific ways (Hao et al., 2022; Man and Jing, 2025). While the core constructs in this study are measured using established psychometric instruments, their enactment and subjective meaning are hypothesized to be culturally situated, necessitating qualitative inquiry into these nuances. There is a critical need for integrative, mixed-methods research that can achieve both statistical precision and phenomenological depth in explaining these pathways.

To address these limitations, the present study employed a convergent parallel mixed-methods design to investigate the mediating role of cognitive reappraisal in the relationship between gratitude, savoring beliefs, and emotional distress among Chinese university students. By simultaneously testing a Structural Equation Model (SEM) and conducting in-depth semi-structured interviews, this research sought to elucidate both the statistical pathways and the underlying subjective experiences of resource-based regulation. By triangulating these data, the study aimed to validate the hypothesized mediation model and determine if the lived experience of students mirrors the proposed sequence of trait-to-regulation-to-outcome. Specifically, we hypothesized that gratitude and savoring would not only directly reduce emotional distress but also indirectly mitigate it by enhancing students’ capacity for cognitive reappraisal. By integrating quantitative and qualitative insights, this study aims to provide a comprehensive understanding of how positive psychological resources facilitate resilience in high-pressure academic environments, ultimately informing the design of culturally sensitive mental health interventions.

## 2 Literature review

### 2.1 The protective role of positive psychological resources: gratitude and savoring

Within the psychological literature, gratitude is conceptualized as both a transient emotional state and a stable dispositional trait characterized by a generalized tendency to appreciate the positive aspects of life and attribute beneficial outcomes to external sources (Emmons and McCullough, 2003; McCullough et al., 2002; Wood et al., 2010). This dispositional framework comprises four distinct facets—intensity, frequency, span, and density—which collectively capture individual differences in grateful affect (McCullough et al., 2002). Complementing the acknowledgment of received benefits, savoring beliefs represent a metacognitive capacity to attend to, appreciate, and enhance positive experiences (Bryant, 1989, 2003; Bryant and Veroff, 2007). Unlike general trait affect, savoring is viewed as a cognitive-affective skill set that allows individuals to up-regulate positive emotions across three temporal orientations: anticipation of the future, active appreciation of the present, and reminiscence of the past (Bryant, 2003; Bryant et al., 2011). Together, these resources promote a balanced time perspective, enabling individuals to maximize present life satisfaction by integrating past successes with future goals (Gao et al., 2025).

Theoretically, gratitude is positioned as a moral affect that reinforces social reciprocity and a core character strength that fosters well-being while buffering against psychopathology (Emmons and McCullough, 2003; Watkins, 2014). Empirical evidence consistently links high levels of dispositional gratitude and savoring capacity to superior mental health outcomes, including heightened life satisfaction and resilience, alongside significantly reduced symptoms of anxiety, depression, and perceived stress (Hurley and Kwon, 2012; Jans-Beken et al., 2020; Lin, 2015; Quoidbach et al., 2010; Smith and Bryant, 2017; Wood et al., 2008; Wood et al., 2010). These protective functions are particularly evident during global crises; for instance, gratitude facilitates adaptive coping mechanisms that mitigate pandemic-related distress (Wolfe, 2021), while savoring prevents high-arousal negative emotions—such as anger and annoyance—from escalating during stressful encounters (Doorley and Kashdan, 2021). The development of these resources may be rooted in an optimistic attributional style regarding positive life events and is further facilitated by trait emotional intelligence, which enhances an individual’s ability to process their emotional landscape (Grandchamp et al., 2021; Torrelles-Nadal et al., 2024).

Beyond psychological adjustment, the influence of these constructs extends to behavioral and physical health domains. Gratitude has been serially linked to improved sleep quality through the enhancement of health self-efficacy (Altier et al., 2025), while savoring interventions have been shown to increase help-seeking intentions among those experiencing depressive symptoms (Straszewski and Siegel, 2018). Furthermore, the efficacy of savoring as an intervention target is supported by meta-analytic evidence demonstrating moderate-to-large reductions in negative emotional symptoms and meaningful improvements in positive psychological states across diverse populations (Chen et al., 2026). While both gratitude and savoring interventions are effective, their impact can be specific to the strategy employed; for example, present-moment attention primarily elevates immediate positive affect, whereas social sharing of positive experiences is a stronger predictor of long-term life satisfaction (Quoidbach et al., 2010; Salces-Cubero et al., 2019). These processes often follow a “Mindfulness-to-Meaning” pathway, where mindfulness-based traits translate into enhanced life satisfaction through the simultaneous mediating roles of gratitude and savoring beliefs (Cheung and Lau, 2021; Cheung and Ng, 2020).

In collectivistic cultural contexts, such as Mainland China, the benefits of gratitude and savoring are deeply intertwined with relational harmony, social support, and filial piety (Hao et al., 2022; Kong et al., 2015; Lin, 2015; Man and Jing, 2025). These resources are especially salient for Chinese university students navigating high-pressure academic environments (Man and Jing, 2025; Zhang et al., 2022). Specifically, higher gratitude and savoring beliefs are associated with lower academic burnout, a greater sense of life meaning, and a reduced risk of suicidal ideation by buffering the impact of chronic academic stress (Li et al., 2012; Lin, 2022). Additionally, the interaction between these resources is critical; savoring appears to offer the greatest relative benefit for anxiety alleviation among students who are less adept at managing negative emotions (Chiu et al., 2020). Despite this evidence, the precise mechanisms through which these positive resources confer protection—particularly the mediating role of cognitive reappraisal—remain underexplored, a gap the current investigation aims to address.

### 2.2 Cognitive reappraisal as a core emotion regulation strategy

At the theoretical level, cognitive reappraisal is defined as an antecedent-focused emotion regulation strategy involving the cognitive reframing of a situation’s meaning to alter its emotional impact (Gross, 1998a, 2015; McRae and Gross, 2020). According to the influential process model of emotion regulation, this strategy is distinct because it is deployed early in the emotion-generative process, well before the emotion response tendency becomes fully activated (Gross, 1998b; Gross and Thompson, 2007). This temporal advantage distinguishes reappraisal from response-focused strategies, such as expressive suppression, which only modifies behavioral expression after an emotion has already been generated (Gross and John, 2003; Gross and Levenson, 1997). Consequently, empirical evidence generally supports the superiority of antecedent-focused strategies, which are more robustly associated with favorable well-being outcomes than response-modulation techniques, particularly among individuals with high emotional intelligence (Schutte et al., 2009). While suppression often leads to increased psychological distress and impaired social connection, reappraisal is consistently linked to enhanced interpersonal functioning and positive affect (Gross and John, 2003; John and Gross, 2004; Webb et al., 2012). Furthermore, daily diary evidence suggest that these strategies play complementary roles; while reappraisal manages negative emotional variance, savoring acts as a buffer that prevents stressors from escalating into high-arousal negative emotions like anger and annoyance (Doorley and Kashdan, 2021).

The psychological benefits of habitual reappraisal are well-documented, showing a strong link to overall psychological well-being and a consistent inverse correlation with symptoms of anxiety and depression (Aldao et al., 2010; Dryman and Heimberg, 2018; Hu et al., 2014; Schafer et al., 2017; Webb et al., 2012). Developmentally, the frequency of reappraisal use remains relatively stable across adolescence, making it a reliable target for long-term health promotion despite the common instability of well-being during early life (Shum et al., 2025). Beyond its cross-sectional associations, longitudinal data position reappraisal as a critical resilience factor that attenuates the growth of depressive symptoms following stressful life events (Gross and John, 2003; Troy et al., 2010). Current systematic evidence classifies it as a “proximal” resilience factor that not only moderates the stressor-outcome relationship but also mediates the link between distal resilience resources and mental health (Riepenhausen et al., 2022).

The efficacy of these cognitive processes is further supported by biological and neurophysiological research. Neuroimaging studies show that successful reappraisal engages prefrontal cortical regions to down-regulate amygdala reactivity (Buhle et al., 2014; Etkin et al., 2015; Ochsner and Gross, 2005). Moreover, top-down cognitive regulation is linked to psychophysiological reward processing, specifically the Reward Positivity (RewP) amplitude, which serves as a biomarker for depressive vulnerability (Irvin et al., 2022). In the specifically academic domain, these regulatory skills contribute to reduced burnout, lower examination anxiety, and improved psychological adjustment among university students (Peña-Sarrionandia et al., 2015).

The salience of these regulatory benefits is particularly evident in the Chinese cultural context, where reappraisal buffers the psychological toll of intense academic pressure and high achievement expectations (Hao et al., 2022). While its benefits appear largely universal across different cultures (Ford and Troy, 2019; Matsumoto et al., 2008), its protective power is derived from its ability to foster cognitive flexibility, enhance adaptive problem-solving, and reduce rumination (Garnefski et al., 2001; Gross, 2015; McRae and Gross, 2020; Schafer et al., 2017). Additionally, by fostering equanimity during interpersonal conflicts, reappraisal strengthens the social support networks that provide further protection against distress (English et al., 2013; Lopes et al., 2005). Recent ecological momentary assessments even suggest a reciprocal relationship; individuals experiencing a deficit in positive emotions may naturally increase their use of reappraisal to restore emotional equilibrium (Colombo et al., 2021).

However, researchers have noted that the efficacy of reappraisal is context-dependent, proving most beneficial when stressors are perceived as having some degree of controllability (Ford and Troy, 2019; McRae and Gross, 2020; Troy et al., 2010, 2013). Crucially, the capacity to successfully deploy this effortful strategy may depend upon the prior availability of positive psychological resources (Garland et al., 2010). This suggests that positive traits like gratitude and savoring provide the necessary cognitive and affective foundations for effective reappraisal, a premise that serves as the cornerstone of the mediation model investigated in the present study.

### 2.3 Gratitude, savoring, and cognitive reappraisal: a mediation framework

The theoretical integration of gratitude and savoring as precursors to cognitive reappraisal is predominantly grounded in the broaden-and-build theory and the upward spiral model of positive emotions (Fredrickson, 1998, 2001, 2004; Fredrickson and Joiner, 2002). These frameworks posit that positive affective states expand an individual’s momentary thought-action repertoire, thereby building enduring psychological resources such as cognitive flexibility and adaptive regulatory capacities (Fredrickson, 2001; Fredrickson and Joiner, 2002; Garland et al., 2010; Tugade and Fredrickson, 2007). In this context, gratitude and savoring act as “upstream” resources that generate the positive emotional “undoing” effect and the psychological space necessary for effortful reframing (Fredrickson, 2001; Watkins, 2014; Wood et al., 2008). Specifically, the temporal focus of savoring—through anticipation and reminiscence—facilitates a sense of “decentering,” allowing individuals to adopt a reflective stance that is a prerequisite for effective reappraisal (Bryant and Veroff, 2007; Smith and Hollinger-Smith, 2015).

Beyond cognitive broadening, the link between these constructs is reinforced through motivational and social-cognitive pathways. Gratitude, functioning as a moral affect, promotes a sense of relational security and psychological safety (McCullough et al., 2001). This perceived social support allows individuals to approach stressors with a “challenge” rather than a “threat” orientation, which significantly enhances the likelihood of engaging in cognitive reappraisal (Cohen and Wills, 1985; Lakey and Orehek, 2011; Taylor and Stanton, 2007; Troy et al., 2013). These processes are further supported by neurobiological evidence showing that gratitude and savoring engage brain regions associated with reward processing and cognitive control, such as the prefrontal and anterior cingulate cortices, which are also critical for the execution of reappraisal strategies (Fox et al., 2015; Kyeong et al., 2017). Furthermore, gratitude-based interventions have been shown to strengthen the functional connectivity within these control networks, providing a neural infrastructure that supports more efficient emotion regulation (Kini et al., 2016).

Empirical evidence across diverse cultural settings provides robust, though often indirect, support for this mediation framework. Cognitive reappraisal has been established as a transdiagnostic mediator linking various positive psychological traits—such as mindfulness, optimism, and self-compassion—to reduced symptoms of anxiety and depression (Desrosiers et al., 2013; Diedrich et al., 2014; Feldman et al., 2007; Finlay-Jones et al., 2015). Specific to the variables of interest, experimental and cross-sectional studies indicate that gratitude and savoring not only correlate with higher reappraisal frequency but also facilitate the spontaneous use of reframing during laboratory and daily life stressors (Bryant, 2003; Lambert et al., 2012; Quoidbach et al., 2010; Watkins, 2014). These regulatory processes also yield functional behavioral outcomes, such as increased help-seeking intentions among individuals with elevated depressive symptoms (Straszewski and Siegel, 2018), and facilitate “benefit-finding” by reinterpreting adverse events to identify positive meaning (Helgeson et al., 2006; Tennen and Affleck, 2002; Watkins, 2014; Wood et al., 2008).

In the Chinese academic context, these relationships are further nuanced by specific relational values and mindfulness-to-meaning pathways. Research involving Chinese cohorts has demonstrated that cognitive reappraisal serves as a key mediator through which mindfulness promotes gratitude and savoring (Cheung and Lau, 2021; Cheung and Ng, 2020), eventually leading to enhanced life satisfaction and a broader sense of meaning (Lin, 2022). Significant correlations between gratitude and reappraisal have been repeatedly documented among Chinese university students (Kong et al., 2015; Li et al., 2012; Man and Jing, 2025), highlighting the interactive nature of positive and negative emotion regulation in mitigating anxiety (Chiu et al., 2020). Despite these findings, existing literature remains largely fragmented, often examining these constructs in isolation and relying heavily on cross-sectional quantitative data (Baron and Kenny, 1986; Wood et al., 2010). The present study addresses these limitations by utilizing a mixed-methods design to investigate the statistical pathways and the lived, phenomenological experiences of this mediation within the high-pressure Chinese higher education environment.

### 2.4 The present study: rationale and hypotheses

While the protective functions of gratitude and savoring (Bryant and Veroff, 2007; Jans-Beken et al., 2020; Wood et al., 2010) and the adaptive efficacy of cognitive reappraisal (Aldao et al., 2010; Gross and John, 2003) are well-documented, a significant gap remains in the empirical testing of the mechanisms connecting these constructs. Despite the strong conceptual foundations provided by the broaden-and-build theory and upward spiral models (Fredrickson, 2001; Garland et al., 2010), investigations into cognitive reappraisal as a mediator between these positive resources and emotional distress are surprisingly scarce. Most inquiries continue to examine these variables in isolation (Kong et al., 2015) or utilize cross-sectional designs that fail to capture the inherently process-oriented nature of adaptive regulation (Man and Jing, 2025; Wood et al., 2008). Furthermore, the interaction of these constructs in specialized contexts, such as post-crisis environments where gratitude may be essential to counteract experiential avoidance, remains under-scrutinized (Velasco et al., 2023).

This theoretical disconnect is compounded by a methodological over-reliance on single-method quantitative designs that reduce mediation to statistical abstractions (Hayes, 2018), often at the expense of understanding lived experiences and underlying phenomenological mechanisms (Fetters et al., 2013; Maxwell, 2012). As scholars increasingly argue, statistical models alone struggle to encompass the personal narratives and cultural contexts that define the enactment of emotion regulation (Creswell and Plano Clark, 2018; Ford and Troy, 2019; Matsumoto et al., 2008; Palinkas et al., 2011). This gap is especially pronounced in research involving Chinese university students; although the individual roles of gratitude (Li et al., 2012; Lin, 2015) and reappraisal (Sai et al., 2016) are established in this population, they have largely been studied in parallel. While recent research has mapped chain-mediation effects from reappraisal to life meaning (Lin, 2022), the specific reciprocal influence of savoring within the high-pressure context of Chinese higher education requires further culturally situated investigation (Man and Jing, 2025; Zhang et al., 2022).

Addressing these multifaceted gaps is essential for refining intervention design, which currently emphasizes direct well-being enhancements over the regulatory mechanisms through which these outcomes are achieved (Cregg and Cheavens, 2021; Emmons and McCullough, 2003; Garland et al., 2010; Seligman et al., 2005; Tugade and Fredrickson, 2007). Clarifying the role of cognitive reappraisal as a mediator is therefore critical for developing interventions that help students translate positive affective experiences into the cognitive flexibility necessary for effective regulation (McRae and Gross, 2020; Quoidbach et al., 2015). To resolve these issues, the present study adopts a convergent parallel mixed-methods design (Creswell and Plano Clark, 2018), grounded in the process model of emotion regulation (Gross, 1998a, 1998b, 2015) and the broaden-and-build theory (Fredrickson, 2001, 2004). Quantitatively, we examine a structural equation model to test whether gratitude and savoring beliefs are negatively associated with emotional distress (H1) and positively associated with cognitive reappraisal (H2), while also determining if reappraisal itself relates negatively to distress (H3) and serves as a partial mediator (H4). Complementing this statistical framework, a qualitative strand utilizing semi-structured interviews explores the “black box” of this mediation, capturing the contextual triggers and subjective meanings that quantitative paths often obscure (Braun and Clarke, 2006; Maxwell, 2012). By triangulating these methodologies, the study generates comprehensive meta-inferences regarding the mechanisms linking positive resources to mental health within the demanding Chinese academic landscape (Creswell and Plano Clark, 2018; Fetters et al., 2013).

## 3 Method

This study employed a convergent parallel mixed-methods design (Creswell and Plano Clark, 2018) to investigate the mechanisms underlying emotional distress. This design allowed for the simultaneous collection and analysis of quantitative and qualitative data to provide a comprehensive understanding of how gratitude and savoring influence emotional distress via cognitive reappraisal. The quantitative strand tested a mediation model using Structural Equation Modeling (SEM), while the qualitative strand explored the phenomenological experiences of participants to elucidate the “how” and “why” behind the statistical pathways.

### 3.1 Participants

The study participants comprised a sample of 502 Chinese university students recruited from three large public universities located in Eastern Mainland China through a combination of convenience and snowball sampling. To ensure the integrity of the data, inclusion criteria required participants to be currently enrolled as full-time students at either the undergraduate or graduate level and to possess native-level fluency in Mandarin Chinese. After excluding five multivariate outliers during the data screening process (see Section 4.1), the final analytical sample consisted of 497 participants (*N* = 497). The final quantitative sample reflected a balanced gender distribution, consisting of 248 females (49.9%) and 249 males (50.1%), with a mean age of 20.3 years (*SD* = 1.8; range: 18–25).

Beyond basic demographics, the sample represented a diverse range of academic disciplines, with approximately 42% of students majoring in the Social Sciences and Humanities, 38% in STEM fields, and 20% in the Arts. Given the rigorous academic environment in Mainland China, participants also reported an average of 12.4 years of English language learning experience (*SD* = 2.1), a factor often associated with the high-pressure academic context of Chinese higher education. Furthermore, the majority of the sample (55%) consisted of upper-division undergraduates (juniors and seniors) or graduate students, while the remaining 45% were in their first or second year of study.

Following the completion of the quantitative phase, a qualitative subsample was derived to provide deeper phenomenological insights into the study’s core variables. Utilizing a purposive sampling strategy characterized by maximum variation, 20 participants (*n* = 20) were selected from the initial pool based on their scores on the Hopkins Symptoms Checklist (HSCL-10) and the Gratitude Questionnaire (GQ-6). This selection process ensured that the qualitative data captured a broad spectrum of experiences, ranging from high to low levels of emotional distress and gratitude. This subsample consisted of 11 females and 9 males, with a slightly higher mean age of 20.5 years. To uphold ethical standards and internal validity, all 497 participants provided informed consent prior to data collection, and all qualitative interviewees were assigned pseudonyms to protect their anonymity throughout the investigative process.

### 3.2 Instrumentation

#### 3.2.1 Dispositional gratitude

Participants’ dispositional gratitude was assessed using the Gratitude Questionnaire-Six Item Form (GQ-6) developed by McCullough et al. (2002). This widely utilized self-report instrument measures the intensity, frequency, span, and density of grateful affect. In the present study, the Chinese version of the GQ-6 (Chen et al., 2008) was employed, consisting of six items (e.g., “I have so much in life to be thankful for” and “If I had to list everything that I felt grateful for, it would be a very long list”). Responses were recorded on a 7-point Likert scale ranging from 1 (*strongly disagree*) to 7 (*strongly agree*). After reverse-coding the two negatively worded items, a mean score was calculated, with higher scores indicating a higher propensity to experience gratitude. The scale demonstrated robust internal consistency in this study (*α* = 0.81), consistent with previous validations in Chinese academic settings.

#### 3.2.2 Savoring beliefs

To measure participants’ perceived capacity to derive pleasure from positive experiences, the Savoring Beliefs Inventory (SBI; Bryant, 2003) was administered. The SBI is a 24-item instrument that captures beliefs across three temporal orientations: anticipating (e.g., “I can feel the excitement within me even before a good thing happens”), savoring the moment (e.g., “I know how to make the most of a good time”), and reminiscing (e.g., “I enjoy looking back on happy times from my past”). Participants responded on a 5-point Likert scale (1 = *not true of me* to 5 = *very true of me*). While the SBI allows for subscale analysis, this study utilized the total savoring score to represent a global savoring disposition. The inventory exhibited excellent reliability in the current sample (α = 0.88), providing a stable measure of the participants’ positive emotional regulation through savoring.

#### 3.2.3 Cognitive reappraisal

The Cognitive Reappraisal subscale of the Emotion Regulation Questionnaire (ERQ; Gross and John, 2003) was used to evaluate the participants’ tendency to use antecedent-focused emotion regulation. This subscale consists of six items designed to measure the frequency with which individuals attempt to change their emotional response by reinterpreting the meaning of a stimulus (e.g., “When I want to feel more positive emotion, I change what I’m thinking about”). For this study, we utilized the validated Chinese version of the ERQ (Wang et al., 2007). Items were rated on a 7-point Likert scale (1 = *strongly disagree* to 7 = *strongly agree*). Higher scores reflected a greater habitual use of reappraisal strategies. The subscale yielded a high internal reliability of α = 0.84 in this sample, indicating that the items were conceptually cohesive for the Chinese student population.

#### 3.2.4 Emotional distress

The level of psychological maladjustment was captured using the Hopkins Symptoms Checklist (HSCL-10), a shortened version of the HSCL-25 (Derogatis et al., 1974) validated for rapid screening by Strand et al. (2003). The HSCL-10 comprises 10 items assessing symptoms of anxiety (e.g., “Feeling tense or keyed up”) and depression (e.g., “Feeling hopeless about the future”) experienced during the preceding week. Each item was rated on a 4-point scale: 1 (*not at all*), 2 (*a little*), 3 (*quite a bit*), and 4 (*extremely*). Given the multidimensional nature of emotional distress, we calculated McDonald’s Omega to account for potential violations of tau-equivalence; the result indicated high internal consistency (*ω* = 0.90). A composite score was derived by averaging the items, where higher values denoted greater levels of emotional distress.

### 3.3 Procedure

The research was conducted in strict accordance with ethical guidelines for human participant research and received formal approval from the Institutional Review Board (IRB) of the host university prior to any data collection. To ensure linguistic and conceptual equivalence between the original English instruments and the Chinese context, all scales underwent a rigorous back-translation protocol as recommended by Brislin (1970). This process involved two bilingual doctoral students who independently translated the scales into Mandarin, followed by a third independent translator who back-translated the Mandarin versions into English to identify any semantic drift. Any resulting discrepancies in phrasing or cultural nuance were subsequently resolved through a committee approach involving the research team and a senior professor of psychology, ensuring that the final Chinese version was both psychometrically sound and culturally appropriate.

Before the main data collection commenced, a pilot study was conducted with a small group of 15 university students to assess the clarity of the items and the functionality of the digital interface, leading to minor adjustments in the survey layout. For the primary quantitative phase, data were collected via an encrypted online survey platform (Wenjuanxing), where participants were first required to read and sign an electronic informed consent form. This document explicitly outlined the study’s purpose, the voluntary nature of participation, and the robust confidentiality measures implemented to protect participant identity. To ensure high data quality and mitigate “careless responding,” two attention-check items were embedded within the survey (e.g., “Please select ‘Strongly Agree’ for this item”), and participants were informed that the survey would take approximately 15 min to complete. As an incentive for their time and effort, students who submitted valid responses were given the opportunity to enter a lucky draw for a small electronic gift card.

Following the initial analysis of the quantitative data, the study transitioned into the qualitative phase where 20 participants were purposively invited for in-depth interviews to further explore the statistical trends observed. The semi-structured interview guide was developed specifically for this study based on three sources: (a) the theoretical frameworks guiding the research—namely the broaden-and-build theory (Fredrickson, 2001) and the process model of emotion regulation (Gross, 2015); (b) the key constructs from the quantitative model (gratitude, savoring beliefs, cognitive reappraisal, and emotional distress); and (c) a review of existing qualitative studies on emotion regulation among university students (e.g., Braun and Clarke, 2006; Smith and Hollinger-Smith, 2015). The guide consisted of 12 open-ended questions organized into four domains: (1) experiences of academic stress and emotional distress (e.g., “Can you describe a recent situation that made you feel very stressed or down?”); (2) use of gratitude in daily life (e.g., “When you feel grateful for someone or something, how does that affect your mood or thinking?”); (3) savoring strategies (e.g., “Do you ever try to hold on to or enhance good feelings? How?”); and (4) cognitive reappraisal (e.g., “When something bad happens, do you try to look at it differently? Can you give an example?”). The guide was piloted with two graduate students (not part of the main study) to check clarity and cultural appropriateness, leading to minor wording adjustments. These semi-structured interviews were conducted via WeChat Work, a secured video conferencing software, and typically lasted between 45 and 60 min in a private, digital setting to ensure participant comfort and privacy. The interview guide was designed to be open-ended, focusing on participants’ lived experiences with academic stress and the specific cognitive strategies they employed to “savor” positive moments or “reframe” negative ones. All interviews were conducted in Mandarin to facilitate a natural and nuanced dialogue, audio-recorded with explicit consent, and subsequently transcribed verbatim. To enhance the trustworthiness and credibility of the findings, member checking was performed by sharing thematic summaries with the interviewees for validation before the transcripts were translated into English for final thematic synthesis and integration.

### 3.4 Data analysis

Data analysis followed a multi-staged approach using SPSS 26.0 and AMOS 24.0 to address both statistical and phenomenological dimensions. Preliminary screening confirmed that missing data occurred randomly (Little’s MCAR test) and were handled via the Expectation–Maximization algorithm. After excluding multivariate outliers identified through Mahalanobis distance, univariate normality was confirmed with skewness and kurtosis values within ±2 and ±7, respectively. Furthermore, Harman’s single-factor test mitigated concerns regarding common method bias (CMB), as the primary factor accounted for only 31.4% of the variance, well below the 50% threshold.

The primary hypotheses were tested via structural equation modeling (SEM) following Anderson and Gerbing’s (1988) two-step procedure. First, confirmatory factor analysis (CFA) validated the measurement model, ensuring robust factor loadings and discriminant validity across the latent constructs. The structural model’s adequacy was subsequently evaluated using established fit indices (*χ*^2^/*df* < 3; *CFI* and *TLI* > 0.90; *RMSEA* and *SRMR* < 0.08). To rigorously examine the hypothesized mediation pathways, we employed a bootstrapping procedure (5,000 resamples), with significance determined by 95% bias-corrected confidence intervals (CIs) excluding zero.

Concurrently, qualitative data were processed using Braun and Clarke’s (2006) thematic analysis. To ensure coding reliability, two researchers (TL and XH) independently coded the first five transcripts (25% of the sample) line-by-line using an initial codebook derived from the interview guide and the quantitative constructs. Inter-coder agreement was calculated using Cohen’s kappa, yielding *κ* = 0.84 for the initial coding phase, indicating excellent agreement (Landis and Koch, 1977). Disagreements were resolved through discussion, and the remaining 15 transcripts were coded by TL, with weekly peer debriefing sessions to maintain consistency. The final codebook was organized into three overarching themes. Trustworthiness was maintained through member checking and peer debriefing, which enhanced the credibility and confirmability of the findings.

Finally, consistent with the convergent parallel design, quantitative and qualitative results were synthesized during the interpretation phase using a joint display matrix (see Table 1). The joint display was constructed by: (1) listing each major quantitative finding (i.e., path coefficients and indirect effects from the SEM); (2) identifying the corresponding qualitative theme(s) that addressed the same conceptual relationship; (3) extracting illustrative participant quotes that exemplified the statistical pattern; and (4) writing a meta-inference that integrated both strands. This procedure allowed us to systematically compare whether the lived experiences (qualitative) supported, contradicted, or enriched the statistical pathways (quantitative). Table 1 provides an example of this mapping for the core mediation pathway (gratitude and savoring → cognitive reappraisal → emotional distress). A joint display facilitated a side by side comparison of SEM path coefficients and narrative excerpts, mapping statistical trends onto lived experiences to provide a comprehensive understanding of how gratitude and savoring function as protective resources against emotional distress.

**Table 1:** Example of joint display: mapping quantitative path coefficients to qualitative themes and participant quotes.

Quantitative finding	Standardized coefficient (*β*)	Qualitative theme	Illustrative participant quote	Meta-inference
Gratitude → Cognitive reappraisal (positive)	*β* = 0.38, *p* < 0.001	Theme 1: Gratitude as a cognitive “anchor” for relational reappraisal	Participant 04: “Feeling grateful for my professor’s help made me re-see the rejection as a growth opportunity instead of a failure.”	Grateful students actively use social support to stabilize emotions, which then enables cognitive reframing.
Savoring → Cognitive reappraisal (positive)	*β* = 0.32, *p* < 0.001	Theme 2: Temporal savoring as a mechanism for “decentering”	Participant 12: “Looking at past travel photos during thesis stress helped me realize life is larger than this one paper; the feedback became fixable.”	Savoring (reminiscing/anticipating) creates psychological distance from immediate stressors, facilitating objective reappraisal.
Cognitive reappraisal → Emotional distress (negative)	*β* = −0.48, *p* < 0.001	Theme 3: “Benefit-finding loop”	Participant 15: “Finding the hidden favor in a project delay built grit and kept me calm in front of peers.”	Successful reappraisal reduces distress and, through positive affect, strengthens the tendency to find benefits in future stressors.
Gratitude → Emotional distress (direct effect)	*β* = −0.15, *p* = 0.002	Theme 1 (anchor)	Participant 09: “Thinking of my parents’ sacrifices made the hard course feel like a way to honor them, not just exhausting.”	Gratitude directly lowers distress by providing relational meaning, beyond its indirect effect via reappraisal.

This table exemplifies the integration procedure for four key pathways.

## 4 Results

### 4.1 Preliminary analysis and descriptive statistics

Data were first screened for missing values and outliers. Little’s MCAR test indicated that missing data (approximately 1.2%) were missing completely at random, χ^2^(85) = 92.40, *p* = 0.27; thus, the Expectation–Maximization (EM) algorithm was employed for imputation. Multivariate outliers were identified using Mahalanobis distance with a threshold of *p* < 0.001, resulting in the exclusion of five cases, leaving a final sample of *N* = 497.

As shown in Table 2, univariate normality was confirmed, with skewness values ranging from −0.54 to 0.62 and kurtosis values from −0.82 to 0.91, both well within the recommended range of ±2 and ±7, respectively. Multivariate normality was assessed via Mardia’s coefficient (22.45), which exceeded the criterion of *p* (*p* + 2); however, given the large sample size and the use of maximum likelihood estimation with bootstrapping (5,000 resamples), the analysis proceeded. To address potential Common Method Bias (CMB) inherent in self-report data, Harman’s single-factor test was performed. The results revealed that the first factor accounted for only 31.4% of the total variance, significantly below the 50% threshold, suggesting that CMB was not a pervasive concern in this study.

**Table 2:** Descriptive statistics and zero-order inter-correlations (*N* = 497).

Variable	*M*	*SD*	Skewness	Kurtosis	1	2	3	4
1. Gratitude	5.12	1.04	−0.42	0.85	—			
2. Savoring beliefs	3.84	0.68	0.21	−0.32	0.52***	—		
3. Cognitive reappraisal	4.65	0.92	−0.15	0.12	0.48***	0.45***	—	
4. Emotional distress	1.95	0.58	0.55	0.74	−0.42***	−0.38***	−0.55***	—

***p < 0.001.

### 4.2 The measurement model

Following the two-step approach recommended by Anderson and Gerbing (1988), we first conducted a Confirmatory Factor Analysis (CFA) to validate the measurement model. To maintain model parsimony and reduce random error, item parceling was employed for the Savoring Beliefs Inventory (SBI), using its three established temporal subscales (Anticipating, Moment, Reminiscing) as manifest indicators. For all other constructs, individual items were used as indicators.

The measurement model demonstrated an excellent fit to the data: χ^2^(146) = 262.80, *p* < 0.001; χ^2^/*df* = 1.80; *CFI* = 0.97; *TLI* = 0.96; *RMSEA* = 0.040 (90% CI [0.033, 0.047]); *SRMR* = 0.038. Construct validity was rigorously assessed through factor loadings, Composite Reliability (CR), and Average Variance Extracted (AVE). As detailed in Table 3, all standardized factor loadings were significant (*p* < 0.001) and ranged from 0.68 to 0.89. CR values ranged from 0.82 to 0.91, exceeding the threshold of 0.70, and AVE values ranged from 0.54 to 0.72, exceeding the recommended 0.50. Reliability estimates using McDonald’s Omega were also high, ranging from 0.81 to 0.90. Furthermore, discriminant validity was established as the square root of the AVE for each latent variable was greater than its highest correlation with any other construct (Table 4).

**Table 3:** Psychometric properties of latent constructs.

Construct	Indicators	λ	α	CR	AVE
Gratitude	GQ1–GQ6	0.72–0.85	0.81	0.88	0.58
Savoring beliefs	3 Parcels	0.79–0.89	0.88	0.91	0.72
Cognitive reappraisal	ERQ1–ERQ6	0.68–0.82	0.84	0.86	0.54
Emotional distress	HSCL1–HSCL10	0.70–0.88	0.90	0.89	0.61

λ = Standardized Factor Loadings; α = Cronbach’s Alpha; CR = Composite Reliability; AVE = Average Variance Extracted.

**Table 4:** Discriminant validity matrix.

	1	2	3	4
1. Gratitude	**0.76**			
2. Savoring beliefs	0.52	**0.85**		
3. Cognitive reappraisal	0.48	0.45	**0.73**	
4. Emotional distress	−0.42	−0.38	−0.55	**0.78**

Bold diagonal values represent the square root of the AVE.

### 4.3 The structural model and hypothesis testing

**Figure 1:**
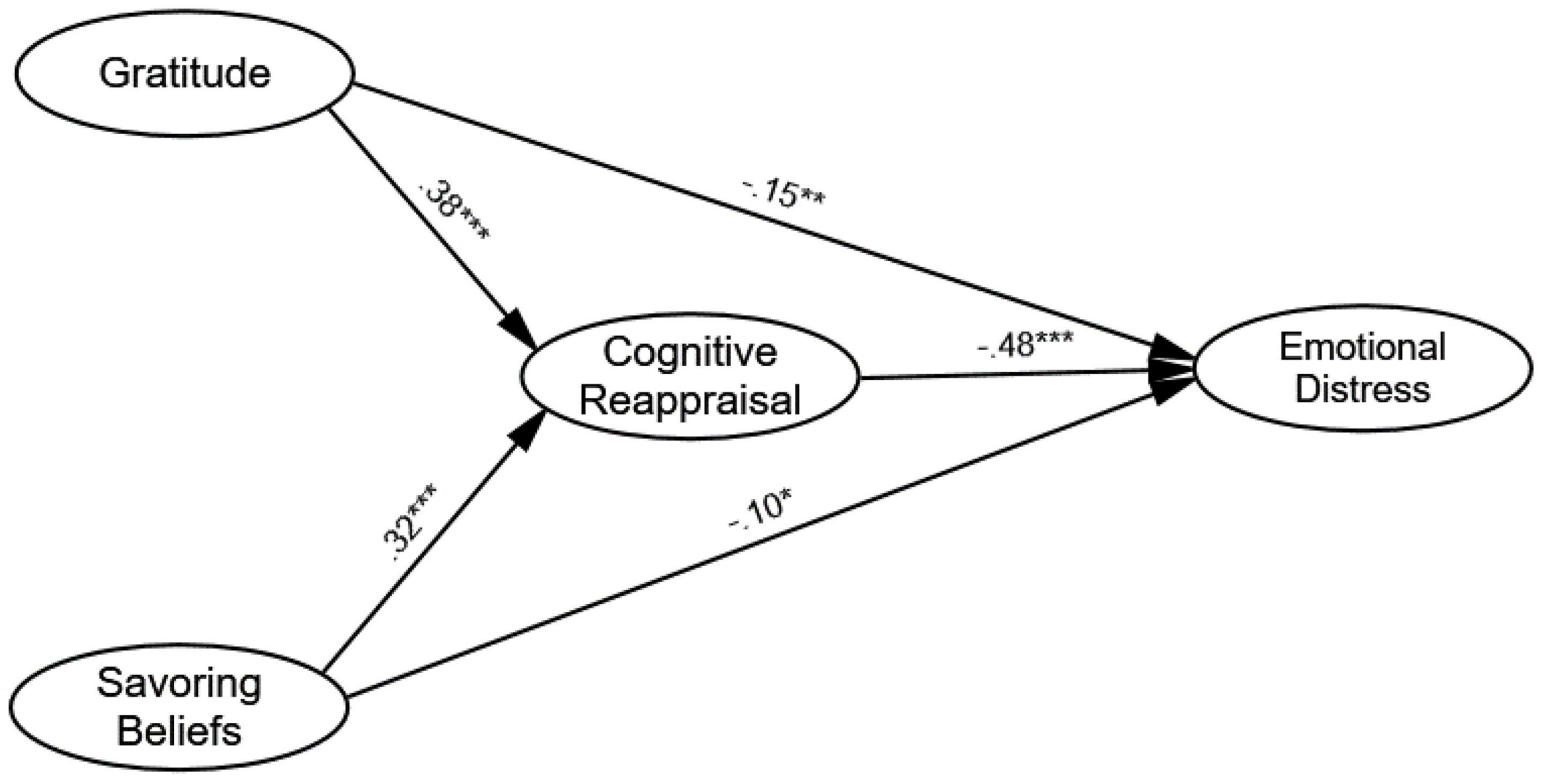
Final structural model of the relationships between gratitude, savoring, cognitive reappraisal, and emotional distress.

The structural model was tested to evaluate the hypothesized relationships between the variables. The model exhibited a strong fit: χ^2^(152) = 273.60, *p* < 0.001; χ^2^/*df* = 1.80; *CFI* = 0.95; *TLI* = 0.94; *RMSEA* = 0.041; *SRMR* = 0.045. The model accounted for a substantial 41% of the variance in emotional distress and 28% in cognitive reappraisal. The standardized path coefficients for the final structural model are illustrated in Figure 1.

Analysis of standardized path coefficients supported the hypothesized positive associations. Specifically, gratitude was a significant positive predictor of cognitive reappraisal (*β* = 0.38, *p* < 0.001), as was savoring beliefs (*β* = 0.32, *p* < 0.001). Regarding direct effects on the dependent variable, gratitude (*β* = −0.15, *p* = 0.002) and savoring beliefs (*β* = −0.10, *p* = 0.015) both maintained significant negative associations with emotional distress. Cognitive reappraisal emerged as the strongest negative predictor of emotional distress (*β* = −0.48, *p* < 0.001).

### 4.4 Mediation analysis

To rigorously test the significance of the indirect effects, a bias-corrected bootstrapping procedure was performed with 5,000 resamples. The results (Table 5) confirmed that cognitive reappraisal partially mediates the influence of both positive traits on emotional distress. The indirect effect of gratitude on distress via reappraisal was significant (standardized indirect effect = −0.18, 95% CI [−0.24, −0.12]). Similarly, the indirect effect of savoring beliefs was significant (standardized indirect effect = −0.15, 95% CI [−0.21, −0.09]). Since both direct and indirect effects remained significant, the findings support a partial mediation model, suggesting that while gratitude and savoring directly alleviate distress, they also function “upstream” to enhance the students’ regulatory capacity to cognitively reframe stressors.

**Table 5:** Standardized direct, indirect, and total effects (bootstrapping 5,000).

Pathway	Direct effect	Indirect effect	Total effect
Gratitude → REAP → ED	−0.15**	−0.18***	−0.33***
Savoring → REAP → ED	−0.10*	−0.15***	−0.26***

ED = Emotional Distress; REAP = Cognitive Reappraisal. *p < 0.05. **p < 0.01. ***p < 0.001.

### 4.5 Qualitative results

The qualitative phase sought to elucidate the “black box” of the statistical mediation, moving beyond path coefficients to understand how Chinese university students experientially navigate the nexus of positive affect and emotional regulation. Following a thematic analysis of 20 semi-structured interviews, three primary themes emerged that provide a process-oriented explanation for why gratitude and savoring function as potent precursors to cognitive reappraisal.

#### 4.5.1 Theme 1: gratitude as a cognitive “anchor” for relational reappraisal

Participants frequently described gratitude not merely as a passive emotion, but as an active cognitive “anchor” that prevented them from spiraling into catastrophic thinking. When faced with high-stakes academic pressure, such as failing a competitive scholarship exam, students reported an initial “tunnel vision” focused on personal inadequacy. However, those with higher gratitude scores utilized their awareness of social support to reframe these setbacks. For instance, Participant 04 (Female, High Gratitude, Low ED) recalled that while she initially “felt like a failure” after a rejected internship application, the shift occurred when she reviewed “messages from my professor who helped me prepare.” By “feeling grateful for her time,” she was able to re-evaluate the outcome as a “growth opportunity” rather than a final judgment of her worth.

This sense of relational capital was echoed by Participant 09 (Male, Moderate Gratitude, Moderate ED), who noted that thinking of his parents’ sacrifices acted as a protective barrier. He explained that instead of viewing a difficult course as a burden, he reframed it as a “way to honor their hard work,” which made the stress feel “meaningful rather than just exhausting.” These narratives support the quantitative finding that gratitude predicts cognitive reappraisal (*β* = 0.38), suggesting that the psychological safety provided by a grateful mindset allows students to move away from “I am useless” self-talk toward a more constructive “I am supported and still learning” perspective.

#### 4.5.2 Theme 2: temporal savoring as a mechanism for “decentering”

The qualitative data revealed that savoring beliefs—particularly reminiscing and anticipating—acted as a buffer that created “psychological distance” from current stressors. This process allowed participants to “step outside” the immediate intensity of their anxiety, a state described by several students as essential for objective reappraisal. Participant 12 (Male, High Savoring, Moderate ED) explained that during his most stressful thesis weeks, looking at travel photos helped him realize that “life is larger than this one paper.” This momentary joy made his academic feedback feel “fixable” rather than “impossible.”

Beyond reminiscing, the act of anticipation served a similar regulatory function. Participant 02 (Female, High Savoring, Low ED) described how looking forward to small weekend rewards created a “mental breathing space” during the week. She noted that when she felt overwhelmed by a deadline, she would intentionally “focus on the excitement” of an upcoming social gathering, which “diluted the bitterness” of her current workload. This suggests that savoring provides the positive emotional “fuel” required for the high-effort cognitive work of reappraisal. By broadening their temporal focus, students transition from reactive distress to a more proactive mode of problem-solving.

#### 4.5.3 Theme 3: the “benefit-finding” loop in reappraisal

A critical insight from the interviews was the description of a self-reinforcing cycle, which we term the “benefit-finding loop.” This loop operates as follows: (a) gratitude and savoring generate positive affect and cognitive flexibility; (b) these states enable the individual to engage in cognitive reappraisal (i.e., reframing a stressor as containing a hidden benefit or learning opportunity); (c) the successful reappraisal produces further positive affect and a sense of mastery; and (d) this enhanced positivity then primes the individual to notice and appreciate positive elements in future challenging situations, thereby closing the loop. Participants identified a specific strategy of “grateful reframing,” where they sought a “silver lining” to maintain personal “face” and social harmony—a culturally salient coping mechanism. Participant 15 (Male, High Gratitude, Moderate ED) described this as “finding the hidden favor” in a negative event. He shared that when his research project was delayed, he reframed the setback as an opportunity to “build more grit,” noting that being thankful for the challenge helped him “stay calm in front of his peers.”

In contrast, students who struggled with this integration found reappraisal nearly impossible. Participant 17 (Female, Low Savoring, High ED) admitted that when her grades dropped, she found it “hard to find anything to be thankful for,” causing her mind to simply “repeat the bad news.” She observed that her more resilient classmates were those who could “find a small thing to appreciate even on a bad day,” suggesting that gratitude serves as the raw material for successful cognitive restructuring. This highlights the qualitative nuance behind the partial mediation found in the SEM: cognitive reappraisal is a powerful tool, but it is significantly more difficult to execute in the absence of the cognitive resources provided by a grateful and savoring mindset.

Overall, integrating these findings with the quantitative model reveals that gratitude and savoring function as antecedent cognitive resources. Statistically, they reduce emotional distress directly, but qualitatively, we see that their primary power lies in their ability to “soften” rigid, negative cognitive schemas. By providing a sense of relational support and an expanded temporal perspective, these constructs allow students to “unstick” themselves from distress. This integrated view suggests that positive psychological traits do not merely replace negative emotions but provide the necessary cognitive flexibility to transform distressing academic experiences into manageable challenges.

## 5 Discussion

The present study employed a convergent parallel mixed-methods design to investigate the complex interplay between gratitude, savoring, cognitive reappraisal, and emotional distress among Chinese university students. By integrating Structural Equation Modeling (SEM) with in-depth thematic analysis, this research sought to move beyond simple correlational patterns to elucidate the psychological and phenomenological mechanisms through which positive psychological resources relate to the mitigation of maladjustment. The quantitative results provided robust support for all hypothesized relationships, revealing that cognitive reappraisal serves as a significant partial mediator, while the qualitative findings offered a granular understanding of these pathways by identifying specific processes such as relational anchoring and temporal decentering. Moving beyond these overarching statistical patterns, the quantitative findings confirmed that both dispositional gratitude and savoring beliefs are significantly and negatively associated with emotional distress. This outcome aligns with a substantial body of extant literature positioning these constructs as core character strengths that buffer against psychopathology (Jans-Beken et al., 2020; Wood et al., 2010).

Specifically, the negative association between gratitude and distress reflects prior research during global crises indicating that a grateful disposition acts as a vital resilience factor by shifting focus away from burdens toward perceived benefits (Wolfe, 2021). In the unique environment of the high-pressure Chinese academic context, this direct effect likely relates to the culturally salient role of gratitude in maintaining interpersonal harmony and filial piety; students who recognize the sacrifices made by their families and mentors may experience a sense of “relational capital” that is associated with lower feelings of isolation and hopelessness (Hao et al., 2022; Man and Jing, 2025). Further supporting this protective role, the significant negative link between savoring and emotional distress corroborates findings that the capacity to up-regulate positive affect is a potent predictor of lower depressive symptoms and stress (Hurley and Kwon, 2012; Smith and Bryant, 2017). Such a connection suggests that savoring—unlike general trait affect—represents a proactive skill set that allows students to mentally “buffer” the rigors of university life by maintaining a “balanced time perspective” (Gao et al., 2025), which aligns with a reduced likelihood of the “tunnel vision” characteristic of anxiety and depression.

A core contribution of this work involves the confirmation that gratitude and savoring are robust positive predictors of cognitive reappraisal. The fact that these variables accounted for significant variance in students’ tendency to use antecedent-focused regulation strategies is conceptually grounded in the broaden-and-build theory, which posits that positive affective states expand an individual’s momentary thought-action repertoire (Fredrickson, 2001). In this sense, gratitude and savoring serve as “upstream” cognitive resources that facilitate the “undoing effect” necessary to disengage from negative stimuli and generate alternative, more adaptive interpretations (Garland et al., 2010). These statistical pathways were notably enriched by qualitative data showing that students do not use reappraisal in a vacuum but rather utilize gratitude as a cognitive “anchor.” For instance, when faced with academic setbacks, grateful students described a process where the acknowledgment of social support stabilized their emotional state, providing the psychological safety required to engage in effortful reframing (McCullough et al., 2001; Watkins, 2014). This pattern aligns with relational regulation theory, suggesting that perceived support—validated through gratitude—facilitates more effective self-regulation (Lakey and Orehek, 2011). Similarly, the qualitative theme of temporal decentering provides a process-oriented explanation for the savoring-reappraisal link by demonstrating how “stepping outside” the immediate stressor through positive reminiscence helps students achieve the “psychological distance” necessary to re-evaluate their challenges objectively (Smith and Hollinger-Smith, 2015).

Beyond these individual paths, the mediation analysis revealed that cognitive reappraisal significantly partially mediated the relationships between the two positive resources and emotional distress. The resulting indirect effects suggest that a substantial portion of the protective power inherent in gratitude and savoring is consistent with the enhancement of regulatory skills, thereby confirming that cognitive reappraisal is a “proximal” resilience factor that is linked to the translation of distal traits into tangible mental health outcomes (Riepenhausen et al., 2022). These findings point toward the existence of a “Benefit-Finding” Loop wherein gratitude and savoring provide the raw materials such as positive affect and cognitive flexibility that serve as the substrate for reappraisal. This pathway is particularly relevant in the Chinese context as it aligns with the cultural emphasis on maintaining “face” (mianzi) and social equanimity. For example, students who can “find the hidden favor” use reappraisal not just to feel better, but to maintain their social standing and continue their academic pursuits with “grit” (Li et al., 2012), an observation that reinforces the “Mindfulness-to-Meaning” pathway where the capacity to reframe adverse events relates to the lowering of symptoms of anxiety and depression (Cheung and Ng, 2020; Lin, 2022).

When evaluating these results, it is essential to consider the specific environment of Mainland Chinese higher education, where academic achievement is a primary source of both identity and stress (Zhang et al., 2022). The robust model fit indicates that the hypothesized relationships are stable within this cohort, suggesting that for Chinese students, cognitive reappraisal is not merely a self-help tool but a culturally adaptive response that balances the pressure of high parental expectations with personal well-being. Because savoring offers the greatest benefit to those less adept at managing negative emotions (Chiu et al., 2020), these positive psychological resources likely act as a “compensatory mechanism” in the face of intense environmental demands. This adaptive function is also evident in the neurophysiological underpinnings of these findings, as top-down regulation associated with gratitude and savoring likely strengthens the functional connectivity within frontoparietal control networks (Kini et al., 2016). Ultimately, by relating to “Reward Positivity,” these positive traits counteract the biological vulnerability to depression that many students face during transition periods (Irvin et al., 2022). Taken together, the findings provide a comprehensive meta-inference: while gratitude and savoring are associated with lower emotional distress, their primary value is reflected in their ability to “soften” rigid, negative cognitive schemas. Indeed, by providing a sense of relational support and an expanded temporal perspective, these constructs enable students to effectively utilize cognitive reappraisal to transform distressing academic experiences into manageable, growth-oriented challenges. Therefore, this research underscores that promoting mental health in university settings requires a dual focus on fostering positive affective resources while simultaneously building the specific regulatory skills necessary to apply those resources to daily stressors.

## 6 Conclusion and implications

This research underscores that the mitigation of emotional distress in Chinese university students is not solely dependent on the absence of negative stressors, but rather on the presence of positive psychological resources that facilitate adaptive cognitive processing. By integrating quantitative path analysis with qualitative phenomenology, the study provides a comprehensive meta-inference: gratitude and savoring serve as foundational cognitive-affective resources that are associated with successful emotion regulation. The findings establish that these positive traits do not merely coexist with stress; they appear to provide the psychological safety and temporal perspective necessary to engage in the high-effort work of cognitive reappraisal. Ultimately, the study reveals that the link between a positive mindset and reduced distress is associated with the specific ability to reframe academic setbacks as growth-oriented challenges. This mechanism is particularly vital in the high-stakes Chinese academic environment, where maintaining social equanimity and personal resilience is a culturally situated necessity.

The results of this study offer several critical contributions to the theoretical landscape of positive psychology and emotion regulation. First, the investigation provides empirical granularity to the Broaden-and-Build theory by identifying cognitive reappraisal as a specific “built” resource associated with the broadening effects of gratitude and savoring, thereby supporting the theory’s application to specific regulatory mechanisms. Furthermore, the study enriches Gross’s Process Model of Emotion Regulation by demonstrating that the efficacy of antecedent-focused strategies is closely associated with the individual’s “cognitive capital.” This finding implies that reappraisal does not operate in a vacuum but is linked to positive affective states. Finally, the research provides a culturally nuanced perspective on the “Mindfulness-to-Meaning” pathway by illustrating how relational values like gratitude and temporal skills like savoring are leveraged by Chinese students to maintain “face” and academic persistence through cognitive reframing.

From a practical standpoint, these findings suggest that university mental health services should move beyond traditional deficit-based interventions and instead incorporate “Resource-Primed Regulation” protocols into their counseling programs. Rather than teaching cognitive reappraisal as a standalone skill, clinicians should consider helping students cultivate a “grateful anchor” or “savoring buffer” to provide the emotional stability required for effective reframing. Such a shift could be implemented through grateful reframing workshops that train students to utilize their recognition of social support as a direct tool for counteracting the “tunnel vision” associated with academic failures. In a similar vein, temporal perspective training could utilize savoring interventions specifically as a “decentering” technique for students experiencing high-arousal anxiety. Structured educational programs offer a viable model for systematically developing these foundational regulatory capacities. For instance, the UNICEF Basic Life Skills Training Program has been shown to improve self-awareness, emotional regulation, and positive thinking among university students in a randomized controlled trial (Sadiq et al., 2026). Adapting such evidence-based life skills frameworks to include gratitude- and savoring-focused modules could provide a scalable, culturally sensitive pathway for implementing the Resource-Primed Regulation approach in Chinese higher education settings. To ensure broad accessibility, future research should investigate whether brief digital micro-interventions (e.g., daily gratitude prompts or savoring exercises) can effectively prime regulatory networks for more adaptive responses.

## 7 Limitations and suggestions for future research

Despite the methodological rigor, several limitations warrant acknowledgment. First, although the study utilized a convergent parallel design to enhance validity, the quantitative data were derived from cross-sectional self-reports. While SEM provided a robust test of the hypothesized mediation, it cannot definitively establish temporal causality. Future research should employ longitudinal or daily diary designs (Ecological Momentary Assessment) to track how fluctuations in gratitude and savoring predict subsequent reappraisal success in real-time. Second, the sample was recruited from universities in Eastern China; given the regional diversity within Mainland China, the findings may not be fully generalizable to students in less urbanized or western regions where academic pressures and social support structures may differ.

Furthermore, while the qualitative strand explored lived experiences, the reliance on interviews may be subject to social desirability bias, particularly regarding culturally sensitive topics like “face” and family expectations. Future studies should consider incorporating objective physiological markers, such as heart rate variability or cortisol levels, to provide a bottom-up biological validation of the “undoing effect” of savoring and gratitude.

Third, the mediation model focused only on cognitive reappraisal, but alternative pathways may also explain the results. For instance, meaning in life mediates the gratitude–mental health link (Adeeb et al., 2026). Gratitude and savoring may reduce distress not only through reappraisal but also by fostering purpose and existential meaning, especially in the Chinese academic context. Future research should compare competing models (e.g., reappraisal vs. meaning in life, or serial mediation). Fourth, the cross-sectional design precludes testing bidirectional relationships. Lower distress may boost gratitude and savoring, and successful reappraisal may increase benefit-finding over time. Longitudinal or experimental designs are needed to test directionality and whether the benefit-finding loop operates as a genuine upward spiral (Fredrickson and Joiner, 2002). Finally, while this study focused on cognitive reappraisal, other regulatory strategies such as acceptance or mindfulness-based distancing remain unexplored in this specific mediation framework. Investigating how positive resources interact with a broader suite of regulation strategies would provide a more holistic understanding of student resilience.

## Data Availability

The raw data supporting the conclusions of this article will be made available by the authors, without undue reservation.
